# Poly[bis­(1-carbamoylguanidinium) [tri-μ-chlorido-dichloridobismuthate(III)]]

**DOI:** 10.1107/S1600536812015668

**Published:** 2012-04-18

**Authors:** Hel Ferjani, Habib Boughzala, Ahmed Driss

**Affiliations:** a Laboratoire de Cristallochimie et des Materiaux, Faculté des Sciences de Tunis, Tunisia

## Abstract

The structure of the title organic–inorganic hybrid compound, {(C_2_H_7_N_4_O)_2_[BiCl_5_]}_*n*_, consists of corrugated chains parallel to [100] of corner-joined [BiCl_6_] octa­hedra, separated by layers of organic 1-carbamoylguanidinum cations. The crystal cohesion is achieved by N—H⋯O and N—H⋯Cl hydrogen bonds, which link the organic and inorganic parts of the structure.

## Related literature

For bis­muth(III) halide organic–inorganic hybrid compounds, see: Masmoudi *et al.* (2011 ▶); Fisher & Norman (1994 ▶); Samet *et al.* (2010 ▶); Papavassiliou *et al.* (1995 ▶); Mousdis *et al.* (1998 ▶); Rhandour *et al.* (2011 ▶). For structures with similar guanidunium cations, see: Bremner & Harrison (2002 ▶, 2003 ▶); Ritchie & Harrison (2003 ▶).
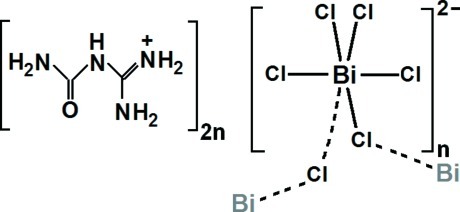


## Experimental

### 

#### Crystal data

(C_2_H_7_N_4_O)_2_[BiCl_5_]
*M* *_r_* = 592.46
Orthorhombic, 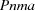

*a* = 7.3795 (8) Å
*b* = 20.706 (4) Å
*c* = 11.028 (2) Å
*V* = 1685.1 (5) Å^3^
*Z* = 4
Mo *K*α radiation
μ = 11.27 mm^−1^
*T* = 298 K
0.53 × 0.25 × 0.17 mm

#### Data collection

Enraf–Nonius CAD-4 diffractometer
Absorption correction: ψ scan (North *et al.*, 1968 ▶) *T*_min_ = 0.048, *T*_max_ = 0.094
2612 measured reflections
1880 independent reflections
1596 reflections with *I* > 2σ(*I*)
*R*_int_ = 0.018
2 standard reflections every 120 min intensity decay: 5%

#### Refinement

*R*[*F* ^2^ > 2σ(*F* ^2^)] = 0.029
*wR*(*F* ^2^) = 0.080
*S* = 1.10
1880 reflections
98 parameters
H-atom parameters not refined
Δρ_max_ = 3.03 e Å^−3^
Δρ_min_ = −1.73 e Å^−3^

### 

Data collection: *CAD-4 EXPRESS* (Duisenberg, 1992 ▶); cell refinement: *CAD-4 EXPRESS*; data reduction: *XCAD4* (Harms & Wocadlo, 1995 ▶); program(s) used to solve structure: *SHELXS97* (Sheldrick, 2008 ▶); program(s) used to refine structure: *SHELXL97* (Sheldrick, 2008 ▶); molecular graphics: *DIAMOND* (Brandenburg, 2008 ▶); software used to prepare material for publication: *publCIF* (Westrip, 2010 ▶).

## Supplementary Material

Crystal structure: contains datablock(s) I, global. DOI: 10.1107/S1600536812015668/bg2453sup1.cif

Structure factors: contains datablock(s) I. DOI: 10.1107/S1600536812015668/bg2453Isup2.hkl

Additional supplementary materials:  crystallographic information; 3D view; checkCIF report

## Figures and Tables

**Table 1:** Hydrogen-bond geometry (Å, °)

*D*—H⋯*A*	*D*—H	H⋯*A*	*D*⋯*A*	*D*—H⋯*A*
N1—H1*A*⋯Cl1^i^	0.86	2.61	3.271 (8)	135
N1—H1*B*⋯Cl2^ii^	0.86	2.50	3.329 (7)	162
N2—H2⋯Cl4^ii^	0.86	2.70	3.524 (7)	160
N3—H3*A*⋯O^iii^	0.86	2.21	3.053 (8)	167
N3—H3*B*⋯O	0.86	2.08	2.734 (8)	132
N4—H4*A*⋯Cl1^iv^	0.86	2.53	3.347 (7)	160
N4—H4*B*⋯Cl4^ii^	0.86	2.59	3.421 (7)	162

Symmetry codes: (i) 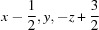; (ii) 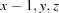; (iii) 
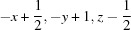
; (iv) 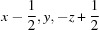.

**Fig. 1.**
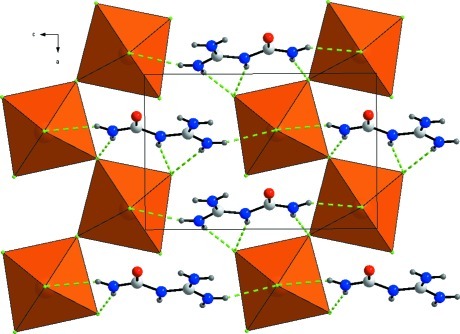
The layred structure of (C2H7N4O)2BiCl5 build up from organic layers, separated by the inorganic 1-D corner-sharing (BiCl5)2- octahedra and showing the N—H···Cl hydrogen bonding (dashed lines).

**Fig. 2.**
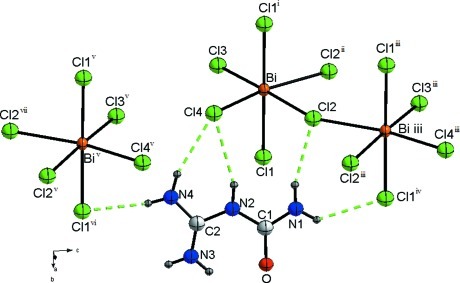
View of the [C2H7N4O]2[BiCl5] with the atom-labelling scheme. Displacement ellipsoids are drawn at the 50% probability level. N-H..O bonds have been omitted for clarity. Symmetry codes: (i): x, 0.5-y, z; (ii): -0.5+x, 0.5-y, 1.5-z ; (iii): 0.5+x, 0.5-y, 1.5-z ;(iv): 0.5+x, y, 1.5-z ; (v): 0.5+x, 0.5-y, 0.5-z ; (vi):0.5+x, y, 0.5-z ;(vii): x, y, -1+z.

## supplementary crystallographic information

### Comment

Recently there has been considerable interest in bismuth (III) halide organic-inorganic hybrid compounds due to their diverse electrical and optical proprieties, as well as their excellent film process ability (Masmoudi *et al.*, 2011; Fisher & Norman, 1994; Samet *et al.*, 2010; Papavassiliou *et al.*, 1995; Mousdis *et al.*, 1998; Rhandour *et al.*, 2011). In particular, the family of bismuth chlorine-based crystals are self-organized low-dimensional nanostructures to form one-,two- or three dimensional networks where BiCl_6_ octahedra can be joined by corners, edges or faces.

We report in this work the synthesis and the structural investigations of the organic-inorganic one-dimensional hybrid compound; Bis(1-carbamoylguanidinum)pentachlorobismuthate(III): [C_2_H_7_N_4_O]_2_[BiCl_5_]. We note that this material was prepared by slow evaporation at room temperature of an aqueous solution containing Bismuth(III) chloride, cyanoguanidine and chlorhydric acid. The abscence of cyanoguanidine in the synthesis result is probably due to their protonation by the chlorhydric acid, giving the 1-carbamoylguanidine cation (protonated guanidineurea or guanylurea).

As shown in Figure 1, the inorganic backbone is stacked as zigzag chains of BiCl_6_ octahedra joined by corner sharing and running along the *a* axis. Organic cations ([C_2_H_7_N_4_O]_2_)^2+^ are located around the inorganic ribbons. Within the BiCl_6_ octahedra the bond lengths around Bi range from 2.546 (3) to 2.880 (3) Å which indicate the dominant ionic character of the Bi—Cl bonds in the inorganic framework. In spite of the notable deviation of the bond angles Cl—Bi—Cl from ideal values of 90° and 180°, the octahedral coordination of bismuth reveals the unstereochemical activity of Bi(III) 6s^2^ lone pair electrons.

The 1-carbamoylguanidinium cations ([C_2_H_7_N_4_O]_2_)^2+^ are approximately parallel to each other (distanced by 3.574 (3) Å), located around the inorganic chains and form stacks oriented along the *a* axis. These planar cations (r.m.s. deviation = 0.0178) have a typical geometrical parameters [d_av_(N—C)= 1.322 Å] as shown in Fig 2, this situation was previously observed in homologous materials involving guanidunium [C_2_H_7_N_4_O] cations (Bremner & Harrison, 2002; Bremner & Harrison, 2003; Ritchie & Harrison, 2003). Strong N—H···Cl hydrogen bonds link the organic part to the inorganic moiety assuming the crystal cohesion.

### Experimental

Bismuth chloride BiCl_3_ and Cyanoguanidine (C_2_H_4_N_4_) (molar ratio 1:2) was dissolved in 20 ml of absolute ethanol with excess of HCl (to improve solubility). The mixture was stirred then kept at room temperature. Three months later, colorless single crystals were obtained and isolated from the reaction. A suitable single-crystal was selected for the structural determination. Supplementary data for this paper are available from the IUCR electronic archives (CCDC number: 866174).

### Refinement

The H atoms on carbon and on nitrogen were placed geometrically and treated as riding on their parent atoms with C—H = 0.96 Å, N—H = 0.86 Å (NH_2_ and NH) with *U*_iso_(H) = 1.2*U*_eq_ (N).

### Crystal data

**Table:**

(C_2_H_7_N_4_O)_2_[BiCl_5_]	*F*(000) = 1112
*M**_r_* = 592.46	*D*_x_ = 2.335 Mg m^−^^3^
Orthorhombic, *P**n**m**a*	Mo *K*α radiation, λ = 0.71073 Å
Hall symbol: -P 2ac 2n	Cell parameters from 25 reflections
*a* = 7.3795 (8) Å	θ = 11–15°
*b* = 20.706 (4) Å	µ = 11.27 mm^−^^1^
*c* = 11.028 (2) Å	*T* = 298 K
*V* = 1685.1 (5) Å^3^	Prism, colourless
*Z* = 4	0.53 × 0.25 × 0.17 mm

### Data collection

**Table:**

Enraf–Nonius CAD-4 diffractometer	1596 reflections with *I* > 2σ(*I*)
Radiation source: fine-focus sealed tube	*R*_int_ = 0.018
Graphite monochromator	θ_max_ = 27.0°, θ_min_ = 2.1°
Non–profiled ω/2θ scans	*h* = −1→9
Absorption correction: ψ scan (North *et al.*, 1968)	*k* = −2→26
*T*_min_ = 0.048, *T*_max_ = 0.094	*l* = −1→14
2612 measured reflections	2 standard reflections every 120 min
1880 independent reflections	intensity decay: 5%

### Refinement

**Table:**

Refinement on *F*^2^	Secondary atom site location: difference Fourier map
Least-squares matrix: full	Hydrogen site location: inferred from neighbouring sites
*R*[*F*^2^ > 2σ(*F*^2^)] = 0.029	H-atom parameters not refined
*wR*(*F*^2^) = 0.080	*w* = 1/[σ^2^(*F*_o_^2^) + (0.0464*P*)^2^ + 4.6267*P*] where *P* = (*F*_o_^2^ + 2*F*_c_^2^)/3
*S* = 1.10	(Δ/σ)_max_ = 0.001
1880 reflections	Δρ_max_ = 3.03 e Å^−^^3^
98 parameters	Δρ_min_ = −1.73 e Å^−^^3^
0 restraints	Extinction correction: *SHELXL97* (Sheldrick, 2008), Fc^*^=kFc[1+0.001xFc^2^λ^3^/sin(2θ)]^-1/4^
Primary atom site location: structure-invariant direct methods	Extinction coefficient: 0.0022 (2)

### Special details

**Table:**

Geometry. All e.s.d.'s (except the e.s.d. in the dihedral angle between two l.s. planes) are estimated using the full covariance matrix. The cell e.s.d.'s are taken into account individually in the estimation of e.s.d.'s in distances, angles and torsion angles; correlations between e.s.d.'s in cell parameters are only used when they are defined by crystal symmetry. An approximate (isotropic) treatment of cell e.s.d.'s is used for estimating e.s.d.'s involving l.s. planes.
Refinement. Refinement of *F*^2^ against ALL reflections. The weighted *R*-factor *wR* and goodness of fit *S* are based on *F*^2^, conventional *R*-factors *R* are based on *F*, with *F* set to zero for negative *F*^2^. The threshold expression of *F*^2^ > σ(*F*^2^) is used only for calculating *R*-factors(gt) *etc*. and is not relevant to the choice of reflections for refinement. *R*-factors based on *F*^2^ are statistically about twice as large as those based on *F*, and *R*- factors based on ALL data will be even larger.

### Fractional atomic coordinates and isotropic or equivalent isotropic displacement parameters (Å^2^)

**Table:**

	*x*	*y*	*z*	*U*_iso_*/*U*_eq_	
Bi	0.62878 (4)	0.2500	0.56736 (2)	0.02190 (13)	
Cl1	0.6373 (2)	0.38017 (8)	0.56635 (13)	0.0404 (4)	
Cl2	0.9594 (3)	0.2500	0.7064 (2)	0.0449 (5)	
Cl3	0.3456 (3)	0.2500	0.4355 (2)	0.0405 (5)	
Cl4	0.8594 (3)	0.2500	0.3856 (2)	0.0388 (5)	
C1	0.1598 (8)	0.4240 (3)	0.5363 (6)	0.0334 (13)	
C2	0.1179 (8)	0.4174 (3)	0.3141 (6)	0.0338 (13)	
O	0.2300 (8)	0.4830 (3)	0.5350 (5)	0.0581 (14)	
N1	0.1374 (9)	0.3932 (3)	0.6381 (6)	0.0543 (17)	
H1A	0.1681	0.4111	0.7054	0.065*	
H1B	0.0918	0.3550	0.6381	0.065*	
N2	0.1010 (8)	0.3947 (3)	0.4317 (4)	0.0376 (13)	
H2	0.0473	0.3581	0.4402	0.045*	
N3	0.1813 (7)	0.4699 (2)	0.2905 (5)	0.0326 (11)	
H3A	0.1882	0.4825	0.2163	0.039*	
H3B	0.2194	0.4946	0.3479	0.039*	
N4	0.0551 (9)	0.3755 (3)	0.2320 (5)	0.0490 (15)	
H4A	0.0580	0.3851	0.1562	0.059*	
H4B	0.0118	0.3389	0.2550	0.059*	

### Atomic displacement parameters (Å^2^)

**Table:**

	*U*^11^	*U*^22^	*U*^33^	*U*^12^	*U*^13^	*U*^23^
Bi	0.02407 (18)	0.02518 (17)	0.01645 (17)	0.000	0.00075 (11)	0.000
Cl1	0.0613 (11)	0.0279 (7)	0.0319 (8)	−0.0047 (7)	−0.0009 (7)	0.0008 (5)
Cl2	0.0457 (13)	0.0501 (12)	0.0388 (12)	0.000	−0.0197 (11)	0.000
Cl3	0.0327 (11)	0.0473 (13)	0.0416 (13)	0.000	−0.0128 (9)	0.000
Cl4	0.0432 (12)	0.0433 (11)	0.0300 (11)	0.000	0.0137 (9)	0.000
C1	0.034 (3)	0.036 (3)	0.030 (3)	0.000 (2)	0.004 (3)	0.002 (3)
C2	0.035 (3)	0.035 (3)	0.031 (3)	0.002 (2)	0.007 (2)	−0.003 (2)
O	0.065 (4)	0.049 (3)	0.060 (3)	−0.005 (3)	−0.003 (3)	0.002 (3)
N1	0.071 (4)	0.058 (4)	0.033 (3)	−0.015 (3)	−0.005 (3)	0.010 (3)
N2	0.052 (3)	0.028 (3)	0.033 (3)	−0.008 (2)	0.003 (2)	0.0019 (19)
N3	0.049 (3)	0.027 (2)	0.022 (2)	−0.010 (2)	0.003 (2)	0.007 (2)
N4	0.071 (4)	0.047 (3)	0.029 (3)	−0.017 (3)	0.008 (3)	−0.007 (2)

### Geometric parameters (Å, º)

**Table:**

Bi—Cl3	2.546 (3)	C2—N3	1.213 (8)
Bi—Cl4	2.630 (2)	C2—N4	1.337 (8)
Bi—Cl1^i^	2.6961 (17)	C2—N2	1.384 (8)
Bi—Cl1	2.6962 (17)	N1—H1A	0.8600
Bi—Cl2^ii^	2.791 (2)	N1—H1B	0.8600
Bi—Cl2	2.881 (3)	N2—H2	0.8600
Cl2—Bi^iii^	2.791 (2)	N3—H3A	0.8600
C1—N1	1.302 (9)	N3—H3B	0.8600
C1—O	1.327 (8)	N4—H4A	0.8600
C1—N2	1.373 (8)	N4—H4B	0.8600
			
Cl3—Bi—Cl4	95.50 (10)	N1—C1—N2	117.9 (6)
Cl3—Bi—Cl1^i^	90.96 (4)	O—C1—N2	121.4 (6)
Cl4—Bi—Cl1^i^	88.95 (3)	N3—C2—N4	124.7 (6)
Cl3—Bi—Cl1	90.96 (4)	N3—C2—N2	122.7 (6)
Cl4—Bi—Cl1	88.95 (4)	N4—C2—N2	112.6 (5)
Cl1^i^—Bi—Cl1	177.28 (8)	C1—N1—H1A	120.0
Cl3—Bi—Cl2^ii^	98.22 (9)	C1—N1—H1B	120.0
Cl4—Bi—Cl2^ii^	166.28 (8)	H1A—N1—H1B	120.0
Cl1^i^—Bi—Cl2^ii^	90.81 (3)	C1—N2—C2	127.5 (6)
Cl1—Bi—Cl2^ii^	90.81 (3)	C1—N2—H2	116.3
Cl3—Bi—Cl2	177.32 (7)	C2—N2—H2	116.3
Cl4—Bi—Cl2	81.82 (10)	C2—N3—H3A	120.0
Cl1^i^—Bi—Cl2	88.99 (4)	C2—N3—H3B	120.0
Cl1—Bi—Cl2	88.99 (4)	H3A—N3—H3B	120.0
Cl2^ii^—Bi—Cl2	84.47 (6)	C2—N4—H4A	120.0
Bi^iii^—Cl2—Bi	148.77 (10)	C2—N4—H4B	120.0
N1—C1—O	120.6 (7)	H4A—N4—H4B	120.0

Symmetry codes: (i) *x*, −*y*+1/2, *z*; (ii) *x*−1/2, *y*, −*z*+3/2; (iii) *x*+1/2, *y*, −*z*+3/2.

### Hydrogen-bond geometry (Å, º)

**Table:**

*D*—H···*A*	*D*—H	H···*A*	*D*···*A*	*D*—H···*A*
N1—H1*A*···Cl1^ii^	0.86	2.61	3.271 (8)	135
N1—H1*B*···Cl2^iv^	0.86	2.50	3.329 (7)	162
N2—H2···Cl4^iv^	0.86	2.70	3.524 (7)	160
N3—H3*A*···O^v^	0.86	2.21	3.053 (8)	167
N3—H3*B*···O	0.86	2.08	2.734 (8)	132
N4—H4*A*···Cl1^vi^	0.86	2.53	3.347 (7)	160
N4—H4*B*···Cl4^iv^	0.86	2.59	3.421 (7)	162

Symmetry codes: (ii) *x*−1/2, *y*, −*z*+3/2; (iv) *x*−1, *y*, *z*; (v) −*x*+1/2, −*y*+1, *z*−1/2; (vi) *x*−1/2, *y*, −*z*+1/2.
